# Inulin Exerts Beneficial Effects on Non-Alcoholic Fatty Liver Disease via Modulating gut Microbiome and Suppressing the Lipopolysaccharide-Toll-Like Receptor 4-Mψ-Nuclear Factor-κB-Nod-Like Receptor Protein 3 Pathway via gut-Liver Axis in Mice

**DOI:** 10.3389/fphar.2020.558525

**Published:** 2020-11-30

**Authors:** Ting Bao, Fang He, Xiaoxia Zhang, Lili Zhu, Zhen Wang, Haixia Lu, Ting Wang, Yiwei Li, Shaoqi Yang, Hao Wang

**Affiliations:** ^1^ Clinical Medical College, Ningxia Medical University, Yinchuan, China; ^2^ Department of Gastroenterology, General Hospital of Ningxia Medical University, Yinchuan, China; ^3^ College of Traditional Chinese Medicine, Ningxia Medical University, Yinchuan, China; ^4^ Department of Pathogenic Biology and Medical Immunology, School of Basic Medical Sciences, Ningxia Medical University, Yinchuan, China

**Keywords:** non-alcoholic fatty liver disease, inulin, nod-like receptor protein 3 inflammasome, macrophage, gut microbiota

## Abstract

**Background:** Non-alcoholic fatty liver disease (NAFLD) is a common metabolic disease worldwide with chronic low-grade inflammation and alteration of gut microbiota. Inulin (INU) has been confirmed to exhibit benefit for metabolic diseases. The aim of this study was to clarify the effects and mechanism of INU on NAFLD inflammation via gut-liver axis.

**Methods:** C57BL/6 mice were randomly divided into four groups: normal diet group (ND); high-fat diet group (HFD); ND with INU group (ND-INU); HFD with INU group (HFD-INU). After 14 weeks of feeding, mice were sacrificed and associated indications were investigated.

**Results:** Significant increases of body weight, liver weight, liver biochemical aspartate aminotransferase, alanine aminotransferase, triglyceride, total cholesterol and pro-inflammatory indicators (Lipopolysaccharide, interleukin (IL)-18, IL-1β, TNF-α and IL-6), as well as a reduction of plasma IL-10 were observed in HFD group, while INU treatment restored these abnormal indicators. The ratio of hepatic macrophages (Mψs) and Toll-like receptor 4^+^ Mψs were both reduced with INU intervention. Nuclear factor-κB, nod-like receptor protein 3, apoptosis-associated speck-like protein and caspase-1 were decreased in HFD-INU group. Additionally, the results of 16S rRNA sequencing and analysis showed that INU administration modulated the composition of gut microbial community in NAFLD mice by up-regulating the abundances of *Akkermansia* and *Bifidobacterium* as well as down-regulating the abundances of *Blautia* and the ratio of *Firmicutes/Bacteroidetes*. Short-chain fatty acids including acetic acid, propionic acid and butyric acid, were increased with INU treatment. Correlation analysis revealed close relationships among inflammatory indicators, metabolic indicators as well as gut microbiota/its metabolite short-chain fatty acids.

**Conclusion:** INU prevents NAFLD via modulating gut microbiota and suppressing Lipopolysaccharide-Toll-like receptor 4-Mψ-Nuclear factor-κB-nod-like receptor protein 3 inflammatory pathway via the gut-liver axis.

## Introduction

Non-alcoholic fatty liver disease (NAFLD) is one of the most common chronic liver diseases worldwide (Abdelmalek, 2016), with 20–30% of a general population prevalence (Younossi et al., 2018). NAFLD represents a disease characterized by hepatocyte steatosis and excessive fat accumulation without abnormal alcohol intake (Ekstedt et al., 2017). NAFLD contributes to increasing the risk of cirrhosis and hepatocellular carcinoma (HCC) (Kanwal et al., 2018), and also closely relates to the progression of cardiovascular diseases, type 2 diabetes and other metabolic diseases (Geurtsen et al., 2019), developing to a major health problem with limited treatment options. Thus, novel strategies for the control of NAFLD are urgently needed.

The gut-liver axis, bidirectional relationship between gut microbiota and liver, is vital for maintaining the homeostasis of gut and liver. Accumulating evidences have demonstrated that gut dysbiosis may be involved in the pathogenesis of NAFLD (Mouzaki et al., 2013; Miura and Ohnishi, 2014; Suk and Kim, 2019). Gut microbiota dysbiosis provokes the damage of intestinal mucosal barriers and enhances intestinal permeability (Luther et al., 2015), leading to translocation of pathogenic bacteria and their metabolites (such as lipopolysaccharide (LPS)) into liver via gut-liver axis for triggering liver chronic inflammation (Zhu et al., 2013). Brandl *et al.* reported that a remarkable reduction of short chain fatty acids (SCFAs) led to dysfunction of gut mucosal barrier in NAFLD (Brandl and Schnabl, 2017). As metabolite end-products of gut bacterial fermentation, SCFAs mainly comprising acetic, propionic and butyric acids, play a critical role in alleviation of glucolipid metabolism, inflammation and gut homeostasis (Clemente et al., 2012). Hence, hepatic chronic inflammation might be relieved by improving the gut microbiota dysbiosis and its metabolites in NAFLD.

Liver is an organ with a variety of immune cells, of which macrophages (Mψs) play a major inflammatory role in the progression of NAFLD (Friedman et al., 2018). Numerous studies have suggested that LPS derived from pathogenic bacteria can translocate into liver and recognize Toll-like receptor 4 (TLR4) of the hepatic Mψs, giving rise to the activation of nuclear factor-κB (NF-κB) and triggering an release of inflammatory cytokines (Luther et al., 2015; Friedman et al., 2018). LPS also can activate NLRP3 inflammasome (a multiproteins compound composed of nod-like receptor protein 3 (NLRP3), apoptosis-associated speck-like protein (ASC) and pro-caspase-1) to motivate the activation of caspase-1 (Pan et al., 2018; Z. Wang et al., 2019). Subsequently, activated caspase-1 promotes the maturation and release of downstream inflammatory cytokines to aggravate the development of NAFLD (Chen et al., 2018). Intriguingly, stimulated NF-κB is crucial for the activation of NLRP3 inflammasome (Qiao et al., 2012). Taken together, enterogenic LPS-mediated activation of TLR4-Mψ-NF-κB-NLRP3 inflammatory pathway may be critical in the development of NAFLD.

Inulin (INU), a kind of indigestible dietary fiber found in a herb *Jerusalem artichoke*, has been reported to show a beneficial effect via regulating gut microbial community (Bindels et al., 2015). Increased studies have shown that INU attenuates metabolic disorders through improving serum lipid, fasting blood glucose and insulin resistance with restoring gut microbiota composition (Zhu et al., 2017; Zou et al., 2018; Misiakiewicz-Has et al., 2019; Song et al., 2019). Our previous studies also demonstrated the amelioration of INU on chronic metabolic diseases (Li et al., 2019; Xue et al., 2019; Yang et al., 2019). However, the effects and mechanism of INU on NAFLD are largely unclear.

In the present study, with a NAFLD model induced by a high-fat diet described previously (Dong et al., 2019), the effects and associated mechanism of INU supplementation on NAFLD inflammation through LPS-TLR4-Mψ-NF-κB-NLRP3 inflammatory pathway were investigated, aiming to enriching theoretical foundation for INU in the prevention and treatment of NAFLD.

## Materials and Methods

### Experimental Animals and Diet

All experiments were approved by the Ethics Committee of General Hospital of Ningxia Medical University (No. 2019-033). Sixty male 4-week-old C57BL/6 mice were obtained from Vital River Laboratory Animal Technology Co., Ltd (Beijing, China). Mice were fed in the laboratory animal research center of Ningxia Medical University (Yinchuan, China) under a 12 h light/dark cycle with free access to food and water. High-fat diet (60% fat, 20% carbohydrate, 20% protein, NO. TP23300) was purchased from TROPHIC Animal Feed High-tech Co., Ltd (Nantong, China). INU was purchased from Fengning Pingan High-tech Industrial Co., Ltd (China).

### Experimental Design

**FIGURE 1:**
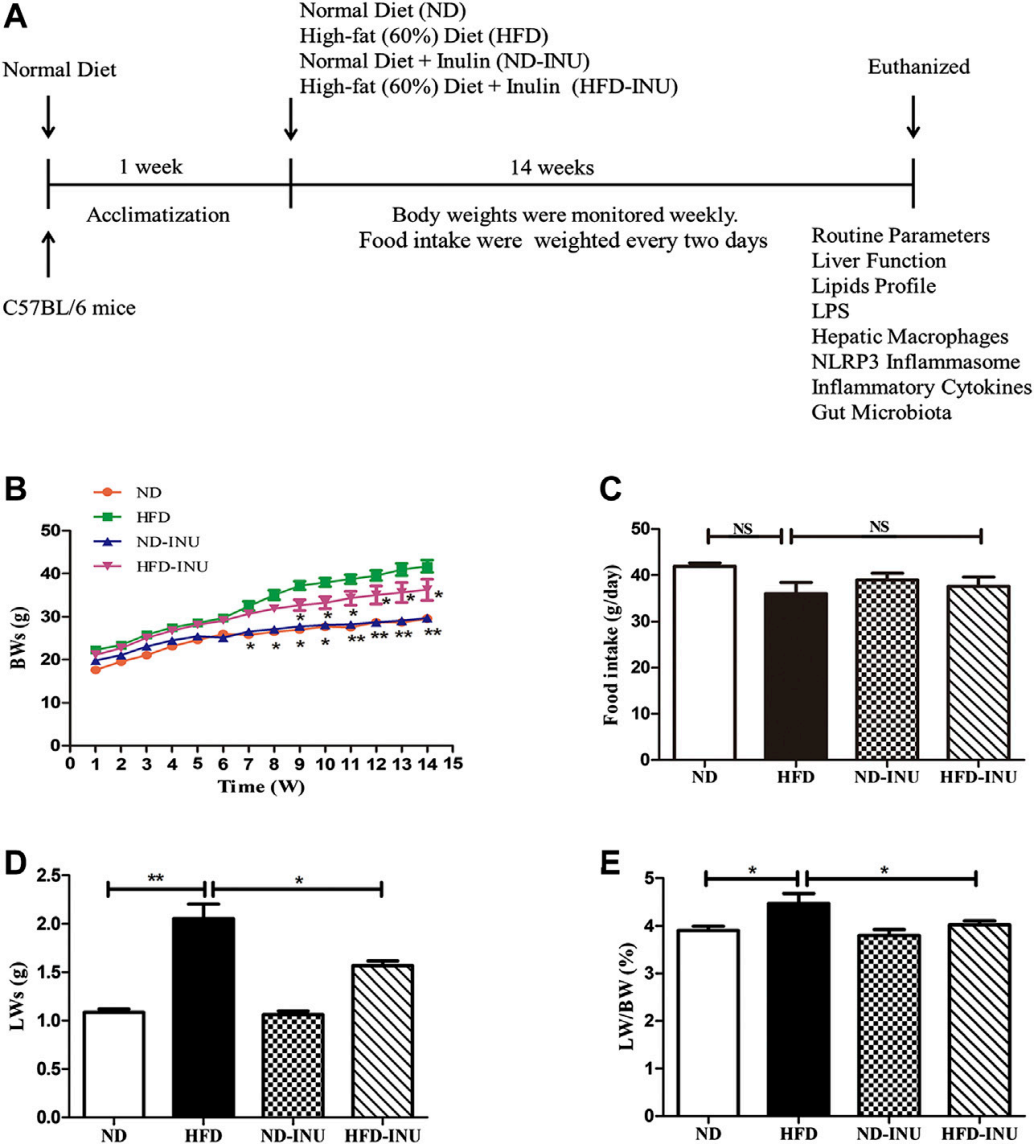
INU treatment improved BW, LW and liver index in HFD-induced NAFLD mice. **(A)** Schematic diagram of the study: C57BL/6 mice with 14–16 g were conditioned for 1 week. For a subsequent 14 weeks, mice in diverse groups were respectively administrated with normal diet and HFD with or without INU. BWs of each group were determined weekly and food intake was recorded every 2 days. At the endpoint of experiment, mice were euthanized and associated indications were investigated. **(B)** BWs of four groups. **(C)** Food intake of four groups. **(D)** LWs of four groups. **(E)** The ratio of LWs to BWs in four groups. Data are expressed as mean ± SEM. *p < 0.05, **p < 0.01, ***p < 0.001 vs. HFD group. BW: body weight; LW: liver weight; INU: inulin; ND: normal diet; HFD: high-fat diet.

As shown in Figure 1A, after 1 week of acclimatization, mice were randomly divided into four groups (15 mice/group): normal diet group (ND), high-fat diet group (HFD), ND with INU group (ND-INU), and HFD with INU group (HFD-INU). Mice in ND and ND-INU groups were fed a normal diet, while HFD and HFD-INU groups were daily given a high-fat diet. Meanwhile, mice in ND-INU and HFD-INU groups were orally administrated with INU (5 g/kg body weight) daily (Zhu et al., 2019). During the experiment, body weights (BWs) of all mice were monitored weekly and food intake was recorded every 2 days. After 14 weeks of feeding, fresh stool samples were obtained and immediately frozen at −80°C for subsequent analysis. At the termination of the experiment, all mice were euthanized by 4% sodium pentobarbital and associated indications were investigated. Blood samples were rapidly collected by orbital bleeding and centrifuged at 4°C (1,200 × *g* for 15 min) to obtain plasma samples, which were stored at −80°C for the further study.

### Plasma Biochemistry Tests

Blood samples were measured for levels of the following biochemical properties: triglyceride (TG), total cholesterol (TC), alanine aminotransferase (ALT) and aspartate aminotransferase (AST) using AU400 automatic biochemical analyzer (Olympus, Japan).

### Oral Glucose Tolerance Test and Homeostatic Model Assessment of Insulin Resistance

Oral glucose tolerance test (OGTT) was carried at week 14 in diverse groups. After 10 h of fasting, blood glucose from one drop of tail blood of each mouse was monitored at five time points (0, 30, 60, 90, and 120 min) after oral administration of glucose (2 g/kg body weight) (Dwiranti et al., 2012). Blood glucose was monitored by a standard glucometer (One Touch Profile, Johnson & Johnson, Inc. Milpitas, CA, United States). Meanwhile, plasma insulin level was quantified by enzyme-linked immunosorbent assay (ELISA) kits (Shanghai Jiang Lai biotech, Shanghai, China) according to the manufacturer’s instructions. homeostatic model assessment of insulin resistance (HOMA-IR) was calculated with the following formula: insulin mIU/L × glucose (mmol/L)/22.5 (Faheem et al., 2020).

### Inflammatory Cytokines

Liver tissues were homogenized and centrifuged at 300 × *g* for 5 min, and the supernatants of homogenates were collected for determination of inflammatory cytokines concentrations. The liver and plasma tumor necrosis factor-α (TNF-α), interleukin (IL)-6, IL-1β, IL-18 and IL-10 were measured respectively by ELISA kits according to the manufacturer’s instructions (Shanghai, Jianglai biotech, Shanghai, China). Optical density was measured at 450 nm within 15 min using an automated microplate reader (Thermo Scientific, United States).

### Plasma and Liver Lipopolysaccharide

Plasma and liver LPS were measured using limulus amebocyte lysate kit (Xiamen Bioendo Technology Co., Ltd, Xiamen, China) according to the manufacturer’s instructions. In brief, 50 µl of plasma or liver tissue homogenate was dispensed to each well in a 96-well plate, respectively. Then 50 μl of limulus amebocyte-like cell lysate reagent was separately added to per well. The plates were incubated at 37°C without light for 30 min. After that, 100 µl of chromogenic substrate was added to each well, and incubation was extended for an additional 6 min at 37°C. Each well was added 100 µl of 25% solution of glacial acetic acid to stop the reaction. Optical density at 562 nm was measured with a microplate reader (Thermo Scientific, United States).

### Histological Analysis and Immunohistochemistry Assay

Liver tissues isolated from mice were immediately fixed in 10% formalin for 24 h and embedded in paraffin. Then paraffin-embedded samples were sectioned at 5 µm thickness and stained with hematoxylin and eosin (H&E). The aggregate NAFLD activity score (NAS) was determined including steatosis (0 point, <5%; one point, 5–33%; two points, 34–66%; three points, >66%), lobular inflammation (0 point, none; one point, <2; two points, 2–4; three points, >4), ballooning (0 point, none; one point, few; two points, many) to assess hepatic steatosis (Brunt et al., 2011).

In immunohistochemical analysis, the sections were deparaffinized and rehydrated through graded alcohol solution, and then liver slides were incubated with 0.1% trypsin-Ethylene Diamine Tetraacetic Acid (EDTA) solution at room temperature for 10 min to unmask antigen. Subsequently, the slides were blocked with 10% normal goat serum for 1 h at room temperature. Samples were incubated with rat anti-mouse F4/80 primary antibody (1:200 dilution, Biolegend, United States) at 4°C for 12 h. After rinsing, the slides were incubated with horseradish peroxidase (HRP)-goat anti-rat IgG (1:500 dilution, Proteintech, China) at room temperature for 1 h. After 3 min of reaction with substrate-chromogen 3, 3’-diaminobenzidine, slides were counterstained with hematoxylin to observe the nucleus. Images were captured with Olympus BX51 microscope (Aomori Olympus, Japan). Positive areas in 20 optical fields (200× magnification) within the liver injury region were then observed. Histological and immunohistochemistry examination for changes were performed in a blinded manner.

### Western Blot

Total proteins of liver tissues were extracted using a commercial kit (Keygen, No. KGP903, China). Protein concentrations were detected with a BCA protein assay kit (Keygen, No. KGBSP002, China). 50 μg protein was subjected to SDS-polyacrylamide gel electrophoresis (SDS-PAGE). Proteins were then transferred to a polyvinylidene difluoride (PVDF) (Millipore, Bedford, MA, United States) membrane electronically. The membranes were blocked with 5% non-fat milk and then incubated with primary antibodies at 4°C overnight as follows: rabbit anti-mouse NLRP3 (1:500 dilution, Abcam, United States), mouse monoclonal ASC (1:500 dilution, Santa Cruz, United States), mouse monoclonal caspase-1 (1:500 dilution, Santa Cruz, United States), rabbit anti-mouse NF-κB (1:500 dilution, Abcam, United States), and mouse monoclonal GADPH (1:1,000 dilution, China). After washing with 1xTBST buffer for three times, membranes were incubated with HRP-conjugated goat anti-mouse antibody (1:1,000 dilution, Abbkine, China) or goat anti-rabbit (1:1,000 dilution, Abbkine, China), respectively. After washing, membranes were visualized with ECL chemiluminescent kit (Thermo Scientific, United States) and measured using the Azure c400 (Thermo Scientific, United States). Images were analyzed using ImageJ software (National Institutes of Health, Bethesda, MD, United States).

### Flow Cytometry Analysis

Mψs derived from mice liver tissues were detected by flow cytometry. Briefly, hepatic tissues (0.1 g) were minced and suspended in 10 ml PBS containing 0.05% (w/v) type Ⅳ collagenase (Sigma, United States) at 37°C for 30 min. Next, specimens were filtered through 200 mesh nylon membrane. Filtered supernatants were washed with RPMI 1640 centrifuging at 50 × *g*, 4°C for 3 min. Supernatants were discarded and pellets were resuspended in 4 ml RPMI 1640. Then, cell suspensions were transferred to 8 ml 50/25% two-step percoll gradient gently in tubes and centrifuged at 500 × *g* for 15 min. After removing the supernatants, the cells were resuspended with 2 ml RPMI 1640 and 5 ml erythrocytes lysis solution. After that, samples were centrifuged for 7 min at 500 × *g*, 4°C. And the final concentration was adjusted to 1×10^6^ cells/ml. To stain Mψs, 1 μl of PE-anti-F4/80 antibody and APC-anti-TLR4 antibody (Biolegend, United States) were simultaneously added in 100 µl of cell suspension incubating on the ice in the dark for 30 min. Finally, these Mψs were measured and analyzed by Beckman Cyto FLEX flow cytometer (Beckman Bioscience, United States).

### Gut Microbiota Analysis

After 14 weeks of intervention, the fresh feces of five mice selected randomly from each group were separately collected and immediately stored at −80°C until DNA extraction. Microbial community genomic DNA was extracted from 0.1 g frozen fecal samples using a TIANamp Stool DNA kit (Tiangen Biotech Co., Ltd, Beijing, China) according to the manufacturer’s instructions. The DNA quality was checked on 1% agarose gel, and DNA purity and concentration were determined with NanoDrop 2000 UV-vis spectrophotometer (Thermo Scientific, Wilmington, United States). The hypervariable region V3-V4 of the 16S rRNA sequence from bacterial DNA samples was amplified using the primers 338F (5’ACT​CCT​ACG​GGA​GGC​AGC​AG-3’) and 806R (5’-GGACTACHVGGGTWTCTAAT-3’) by an ABI Gene Amp^®^ 9700 PCR thermocycler (ABI, CA, United States). The PCR reaction was performed as follows: initial denaturation for 3 min at 95°C, followed by 27 cycles of denaturing at 95°C for 30 s, annealing at 55°C for 30 s and extension at 72°C for 45 s, and then single extension at 72°C for 10 min, finally end at 4°C. PCR reactions were performed in triplicate. Purified amplicons were pooled in equimolar and paired-end sequenced (2 × 300) on an Illumina MiSeq platform (Illumina, San Diego, United States) according to the standard protocols by Majorbio Bio-Pharm Technology Co. Ltd (Shanghai, China).

The raw 16S rRNA gene sequence reads were demultiplexed, quality-filtered, merged and clustered into operational taxonomic units (OTUs) with 97% similarity cutoff, and chimeric sequences were identified and removed.

### Fecal Quantifications of Short-Chain Fatty Acids by Gas Chromatography-Mass Spectrometer

SCFAs (acetic acid, propionic acid and butyric acid) were measured using an Agilent 7890A gas chromatography coupled with an Agilent 5975C mass spectrometric detector (Agilent Technologies, United States). The GC was fitted with a capillary column Agilent HP-INNOWAX (30 m × 0.25 mm i. d. × 0.25 µm) (Agilent Technologies, United States), and helium was used as the carrier gas at 1 ml/min. Injection was made in split mode at 10:1, with an injection volume of 1 µl and an injector temperature of 250°C. The temperature of the ion source, interface, and quadrupole were 230, 250, and 250°C, respectively. The column temperature was initially 90°C and then increased to 120°C at 10°C/min, to 150°C at 5°C/min, and finally to 250°C at 25°C/min; and this temperature was kept for 2 min (total run-time of 15 min). The detector was operated in electron impact ionization mode (electron energy 70 eV) using full scan and single ion monitoring.

### Statistical Analysis

Statistical analysis was carried out using GraphPad Prism, version 6.0 (GraphPad Software Inc., CA, United States). All results are presented as mean ± standard deviation. Once the data showed an equal of Gaussian distribution and variance, differences among multiple comparisons were performed using one-way analysis of variance (ANOVA) followed Tukey’s post hoc test. Otherwise, Kruskal-Wallis test and followed Dunn’s post hoc test were applied. Correlation analysis was performed using Spearman. *p* < 0.05 was considered to be statistically significant.

## Results

### Inulin Treatment Improved BWs, Liver Weights and Liver Index in High-Fat Diet-Induced Non-Alcoholic Fatty Liver Disease Mice

**FIGURE 2:**
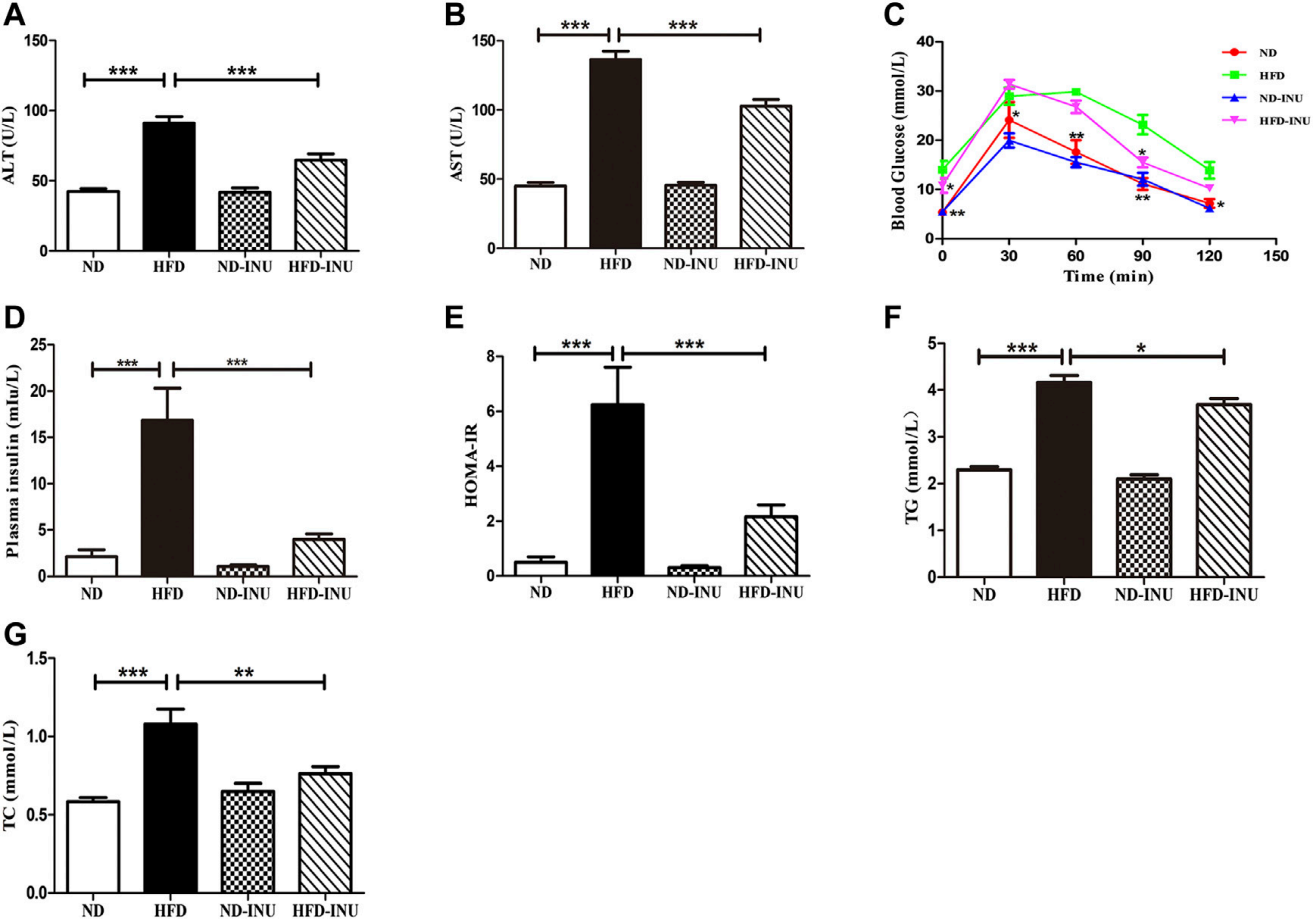
Effects of INU treatment on ALT, AST, TG, TC, OGTT, insulin and HOMA-IR in NAFLD. **(A)** ALT. **(B)** AST. **(C)** OGTT. **(D)** Insulin. **(E)** HOMA-IR. **(F)** TG. **(G)** TC. Data are expressed as mean ± SEM. *p < 0.05, **p < 0.01, ***p < 0.001. All experiments were performed in triplicate. ALT: alanine aminotransferase; AST: aspartate aminotransferase; HOMA-IR: homeostatic model assessment of insulin resistance; OGTT: oral glucose tolerance test; TG: triglyceride; TC: total cholesterol; INU: inulin; ND: normal diet; HFD: high-fat diet.

As expected, at the end of the experiment, BWs (*p* < 0.05, Figure 1B), LWs (*p* < 0.01, Figure1D) and liver index (LWs/BWs) (*p* < 0.05, Figure 1E) in HFD group were dramatically higher than those in ND group. In contrast, INU administration obviously reduced BWs, LWs and liver index in HFD-INU group (*p* < 0.05, Figures 1B,D) compared to HFD group. Moreover, food intake showed no significant difference in HFD-induced groups (*p* > 0.05, Figure 2C), suggesting that the effects of INU on BWs and LWs were not due to influence of energy intake.

### Inulin Alleviated Liver Function and Glucose Metabolism in Non-Alcoholic Fatty Liver Disease

Plasma AST and ALT levels were determined to confirm the liver function. Compared with ND group, increases of plasma ALT and AST (*p* < 0.001, Figures 2A,B) were measured in mice with HFD. Nevertheless, the plasma ALT and AST levels in HFD-INU group were significantly decreased (*p* < 0.001, Figures 2A,B). Due to close correlations of glucose tolerance and insulin resistance with the progression of NAFLD, we examined OGTT and plasma insulin to assess the effect of INU on glucose metabolism. The results showed that the levels of plasma insulin (*p* < 0.001, Figure 2D) and HOMA-IR (*p* < 0.001, Figure 2E) were notably increased in HFD group compared to ND group. Intriguingly, the improvements of OGTT, plasma insulin and HOMA-IR were observed in HFD-INU group (*p* < 0.001, Figures 2C–E), indicating that INU effectively attenuated insulin resistance in NAFLD.

### Inulin Attenuated Lipid Accumulation in Plasma and Liver in Non-Alcoholic Fatty Liver Disease

**FIGURE 3:**
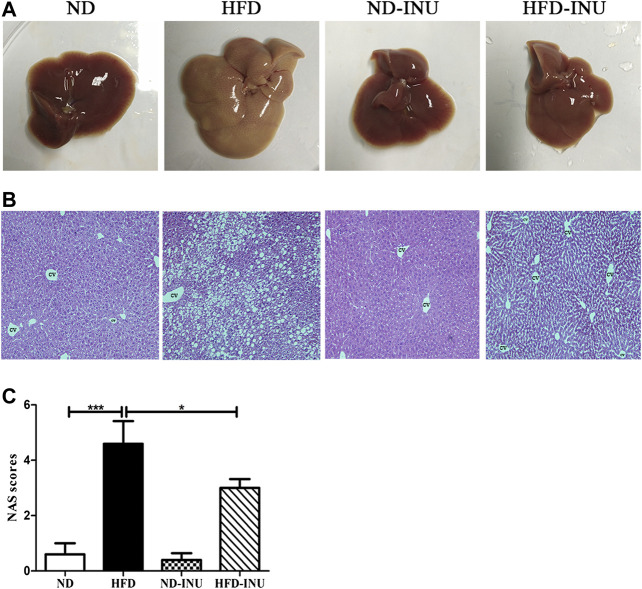
Effects of INU treatment on liver injury in NAFLD. **(A)** Liver appearance. **(B)** Representative images of hepatic hematoxylin and eosin (H&E) staining. **(C)** NAS. Data are expressed as mean ± SEM. *p < 0.05, **p < 0.01, ***p < 0.001. CV: central vein. Original magnification, ×200. All experiments were performed in triplicate. NAS: NAFLD Activity Score; INU: inulin; ND: normal diet; HFD: high-fat diet.

Abnormal lipid accumulation is considered to be crucial in the development of NAFLD. In this study, TG (*p* < 0.001) and TC (*p* < 0.001) concentrations in plasma were significantly elevated in HFD group compared with those in ND group (Figures 2F,G). But these levels were remarkably decreased in HFD-INU group (*p* < 0.05, Figures 2F,G). HFD-induced accumulation of fat in liver appearance was observed in both HFD and HFD-INU groups (Figure 3A). Aggregated hepatic steatosis with HFD was evidently determined by the score of NAS compared to ND group (*p* < 0.001, Figures 3B,C). However, compared to HFD group, the NAS score was significantly decreased in HFD-INU group (*p* < 0.05, Figures 3B,C). Taken together, these results demonstrated that INU attenuated HFD-induced lipid accumulation, indicating the potential application of INU for NAFLD.

### Inulin Reduced Mψs and Toll-Like Receptor 4 Expression of Mψs in Liver in Non-Alcoholic Fatty Liver Disease

**FIGURE 4:**
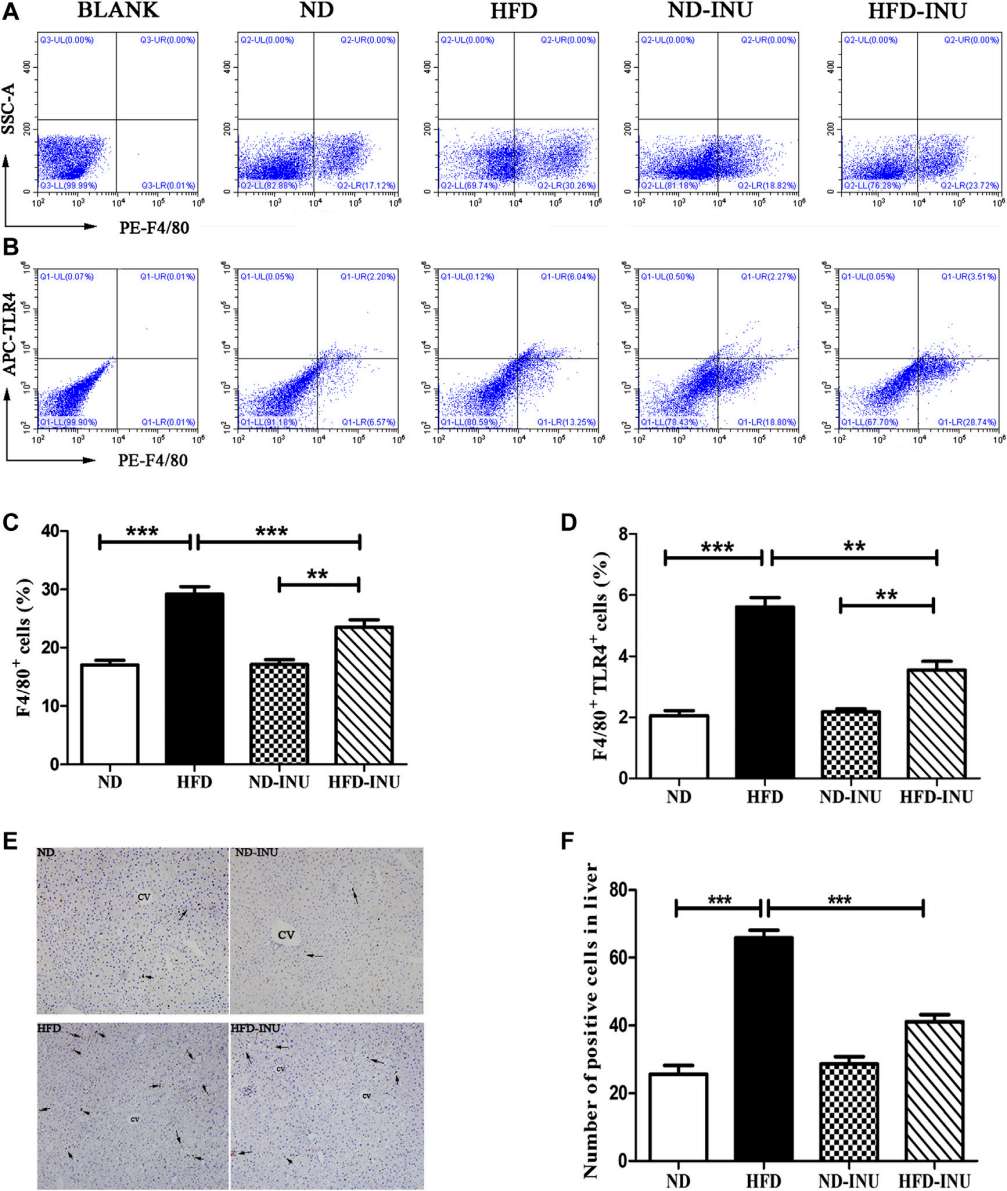
Effects of INU treatment on hepatic Mψs and TLR4 expression of Mψs in NAFLD by flow cytometry and immunohistochemistry. **(A)** Flow cytometry analysis of liver F4/80 ^+^ cells in diverse groups **(B)** Flow cytometry analysis of liver F4/80^+^TLR4^+^ cells in diverse groups **(C)** The proportion of liver F4/80 ^+^ cells **(D)** The proportion of liver F4/80 ^+^ TLR4^+^ cells **(E)** Results of hepatic immunohistochemistry were stained by F4/80 antibody **(F)** The number of positive Mψs in diverse groups. Hepatic Mψs were stained as brown and shown with arrows in the figure **(E)**. CV: central vein. Original magnification, ×200. Data are expressed as mean ± SEM; *p < 0.05, **p < 0.01, ***p < 0.001. All experiments were performed in triplicate. Mψ: macrophage; TLR4: Toll-like receptor four; INU: inulin; ND: normal diet; HFD: high-fat diet.

To further analyze the effects of INU on liver Mψs, hepatic F4/80^+^ and F4/80^+^TLR4^+^ Mψs were measured by flow cytometry (Figures 4A,B). The ratio of hepatic F4/80^+^ cells and F4/80^+^TLR4^+^ cells were increased in HFD group (*p* < 0.001, Figures 4C,D) compared to ND group. However, the proportions of F4/80^+^ cells (*p* < 0.001, Figure 4C) and F4/80^+^ TLR4^+^ cells (*p* < 0.01, Figure 4D) were respectively lower in INU treatment. Immunohistochemistry was further used to determine the effect of INU on F4/80^+^ Mψs (Figure 4E). F4/80^+^ Mψs was elevated in HFD group compared to ND group (*p* < 0.001, Figure 4F). In contrast, after INU supplementation, F4/80^+^ Mψs were reduced (*p* < 0.001, Figure 4F), which was consistent with results of flow cytometry.

### Inulin Suppressed Nod-Like Receptor Protein 3 Inflammasome and Nuclear Factor-κB in Liver of Non-Alcoholic Fatty Liver Disease

**FIGURE 5:**
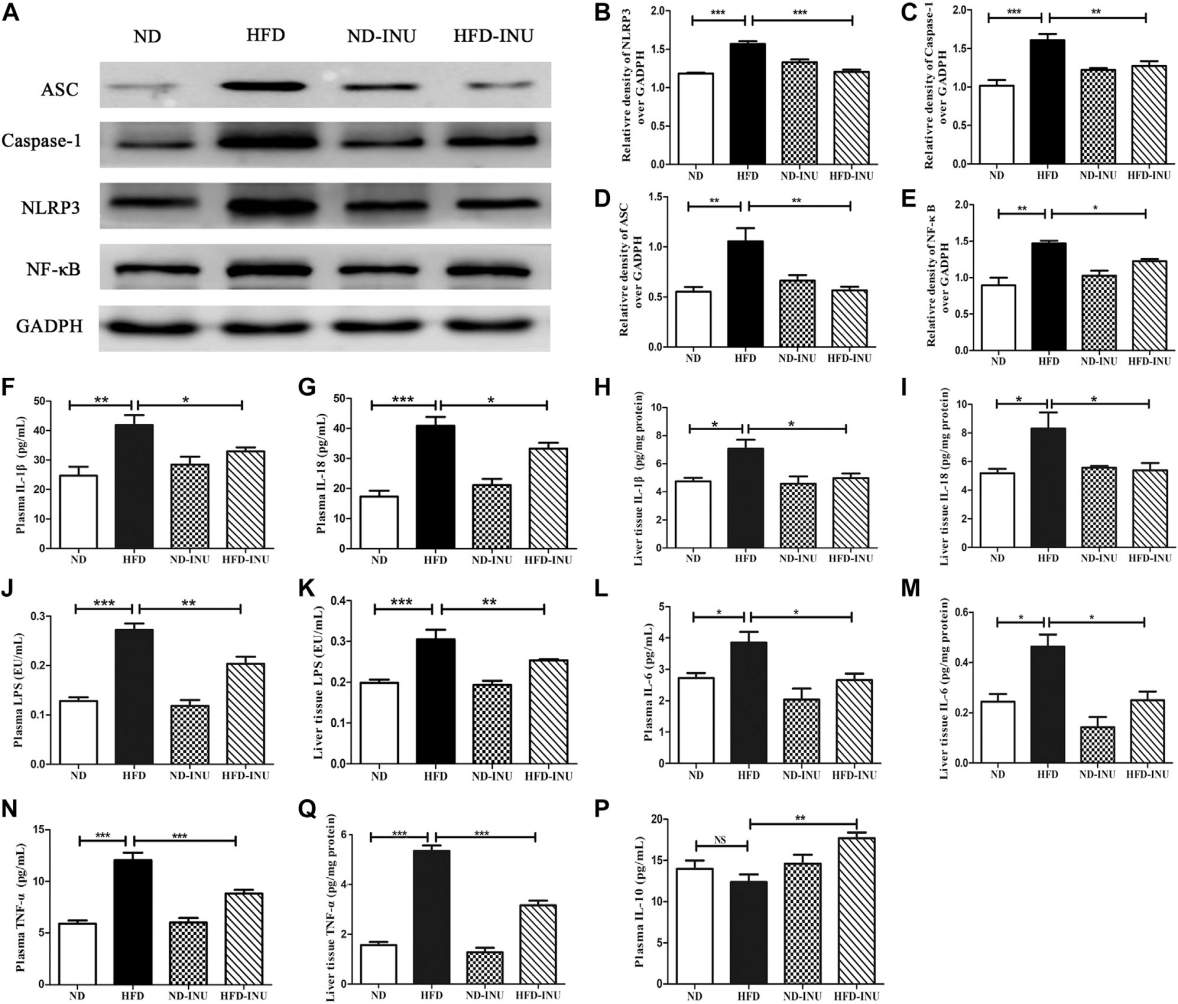
Effects of INU treatment on NLRP3 inflammasome, NF-κB and inflammatory indications in NAFLD. **(A)** Western blot analysis of NLRP3, ASC, Caspase-1 and NF-κB. Semi-quantitative analysis of the relative levels of NLRP3 **(B)**, Caspase-1 **(C)**, ASC **(D)** and NF-κB **(E)** by densitometric analysis. Levels of liver and plasma IL-1β **(F,H)**, IL-18 **(G,I)**, LPS **(J,K)**, IL-6 **(L,M)**, TNF-α **(N,O)** as well as plasma IL-10 **(P)** were determined using ELISA kits, respectively. Data are expressed as mean ± SEM; NS: no significant difference. *p < 0.05, **p < 0.01, ***p < 0.001. All experiments were performed in triplicate. NLRP3: nod-like receptor protein 3; ASC: apoptosis-associated speck-like protein; NF-κB: nuclear factor-κB; IL: interleukin; LPS: lipopolysaccharide; TNF-α: tumor necrosis factor-α; INU: inulin; ND: normal diet; HFD: high-fat diet.

Due to NLRP3 inflammasome and NF-κB pathway play a crucial role in inflammation, we investigated whether anti-inflammatory effect of INU on NAFLD was associated with suppression of NLRP3 inflammasome/NF-κB activation in HFD feeding mice. Western blot was used to determine the expressions of NLRP3, ASC and caspase-1. As a result, HFD significantly increased the expressions of liver NLRP3 (*p* < 0.001, Figures 5A,B), caspase-1 (*p* < 0.001, Figures 5A,C) and ASC (*p* < 0.01, Figures 5A,D) compared with those in ND group, which were obviously decreased in HFD-INU group. In addition, HFD induced a notable increase of NF-κB, but a significant reduction of which was observed in HFD-INU group (*p* < 0.05, Figures 5A,E), suggesting that INU may inhibit the activation of NLRP3 inflammasome depending on NF-κB signaling pathway.

### Inulin Regulated Inflammatory Indications in Plasma and Liver of Non-Alcoholic Fatty Liver Disease

LPS-triggered inflammatory pathway based on gut-liver axis has been thought to contribute to the inflammation in NAFLD (Zhu et al., 2013; Z. Wang et al., 2019). In this study, LPS levels of plasma and liver in HFD group were higher than that in ND group (*p* < 0.01, Figures 5J,K). Increased LPS in plasma and liver was significantly decreased after INU intervention (*p* < 0.01, Figures 5J,K). We further examined concentrations of pro-inflammatory cytokines including IL-1β, IL-18, TNF-α, IL-6 and anti-inflammatory IL-10, respectively. Compared to ND group, pro-inflammatory cytokines in plasma and liver tissue including IL-1β (*p* < 0.01, Figures 5F,H), IL-18 (*p* < 0.01, Figures 5G,I), IL-6 (*p* < 0.05, Figures 5L,M) and TNF-α (*p* < 0.001, Figures 5N,O) were elevated, which were rectified in HFD-INU group. Moreover, a decrease trend of anti-inflammatory IL-10 in plasma was observed in HFD group without significant difference compared with ND, which was obviously enhanced in HFD-INU group (*p* < 0.01, Figure 5P). Taken together, these results demonstrated that the effectiveness of INU on liver chronic inflammation in NAFLD is partially due to the reduction of LPS translocation.

### Inulin Restored Gut Dysbiosis in Non-Alcoholic Fatty Liver Disease Mice

Numerous studies have been increasingly demonstrated to explore the crucial role of gut microbiota in the pathogenesis of NAFLD (Zhu et al., 2013; Luther et al., 2015; Delzenne et al., 2019; Suk and Kim, 2019). To further confirm the effect of INU on gut microbiota in diverse groups, predominant bacterial species at the phylum and genus levels were investigated by 16S rRNA sequencing and analysis.

**FIGURE 6:**
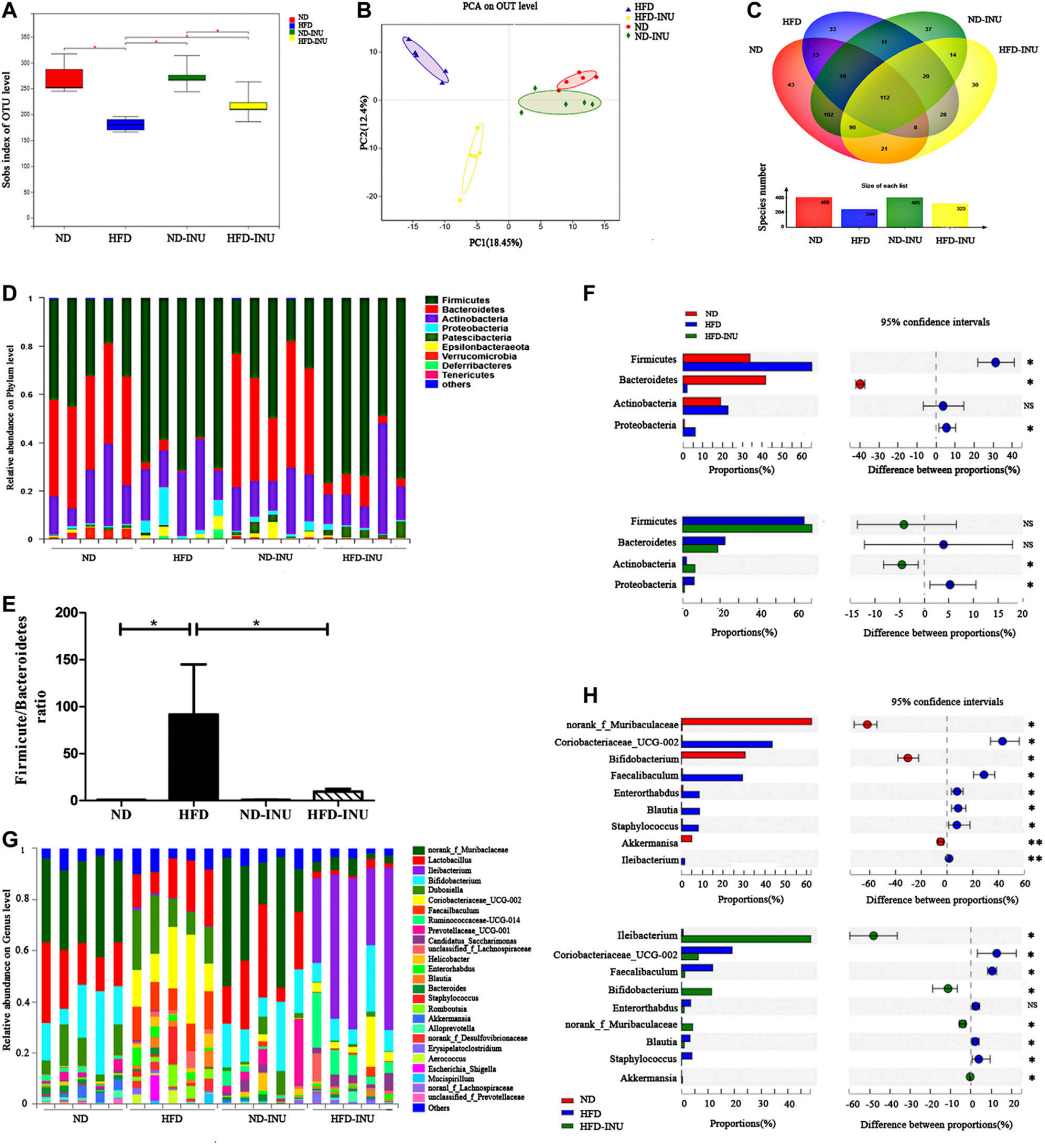
The alteration of gut microbial species from mice feces of diverse groups. **(A)** Sobs index showing difference in terms of abundance and diversity of gut microbiota in diverse groups. **(B)** Principal component analysis (PCA) analysis. **(C)** Venn diagram. **(D,F)** The relative abundance of microbial species in phylum level. **(E)** The ratio of *Firmicutes* to *Bacteriodietes* in phylum level. **(G,H)** The relative abundance of microbial species in genus level. NS: no significant difference. *p < 0.05, **p < 0.01. INU: inulin; ND: normal diet; HFD: high-fat diet.

As shown in Figure 6A of the sobs index based on OTU level, HFD reduced gut bacterial diversity in mice (*p* < 0.05) compared to ND, whereas NAFLD mice featured a markedly increased sobs index after INU treatment (*p* < 0.05). The overall composition of bacterial community was analyzed using Bray-Curtis-based Principal component analysis (PCA) and Venn diagram (Figures 6B,C). We found different clusters in PCA of gut microbiota between HFD and ND groups, as well as distinct clusters in PCA after INU supplementation compared to HFD group (Figure 6B). In parallel, Venn diagram showed significantly different number of gut microbial species in HFD feeding with or without INU supplementation (Figure 6C).

At the phylum level, we found that an obviously increased abundance of *Firmicutes* (*p* < 0.05) and a decreased proportion of *Bacteroidetes* (*p* < 0.05) in HFD group compared to ND group, which were mildly modulated by INU without significant difference (*p* > 0.05, Figures 6D,F). Moreover, increased ratio of *Firmicutes* to *Bacteroidetes* in NAFLD (*p* < 0.05) was restored about 80% by INU supplementation (*p* < 0.05, Figure 6E). Thus, INU had a major influence on the ratio of *Firmicutes* to *Bacteroidetes* under HFD feeding. In addition, INU also partially restored increased abundance of *Proteobacteria* in NAFLD (*p* < 0.05, Figure 6F).

To further evaluate the effect of INU on microbial community in genus level, top 26 genus species were analyzed. Importantly, HFD induced significant reductions of *Bifidobacterium* (*p* < 0.05) and *Akkermansia* (*p* < 0.01) as well as an augmentation of *Blautia* (*p* < 0.05) compared with those in ND group, whereas which was restored after INU treatment (*p* < 0.05, Figures 6G,H). Additionally, a high relative abundance of *Ileibacterium* (*p* < 0.01) was observed in HFD compared to ND group, which strengthened memorably with INU intervention (p < 0.05, Figures 6G,H). Collectively, the genus results showed that HFD consumption changed the initial proportion of OTUs at genus level, mainly including reduced *Bifidobacterium*, *Akkermansia*, as well as increased *Blautia* and *Ileibacterium*. Conversely, INU supplementation restored gut dysbiosis by up-regulating *Akkermansia*, *Bifidobacterium* and down-regulating *Blautia*.

### Inulin Enhanced the Contents of Short-Chain Fatty Acids in Mice Feces

**FIGURE 7:**
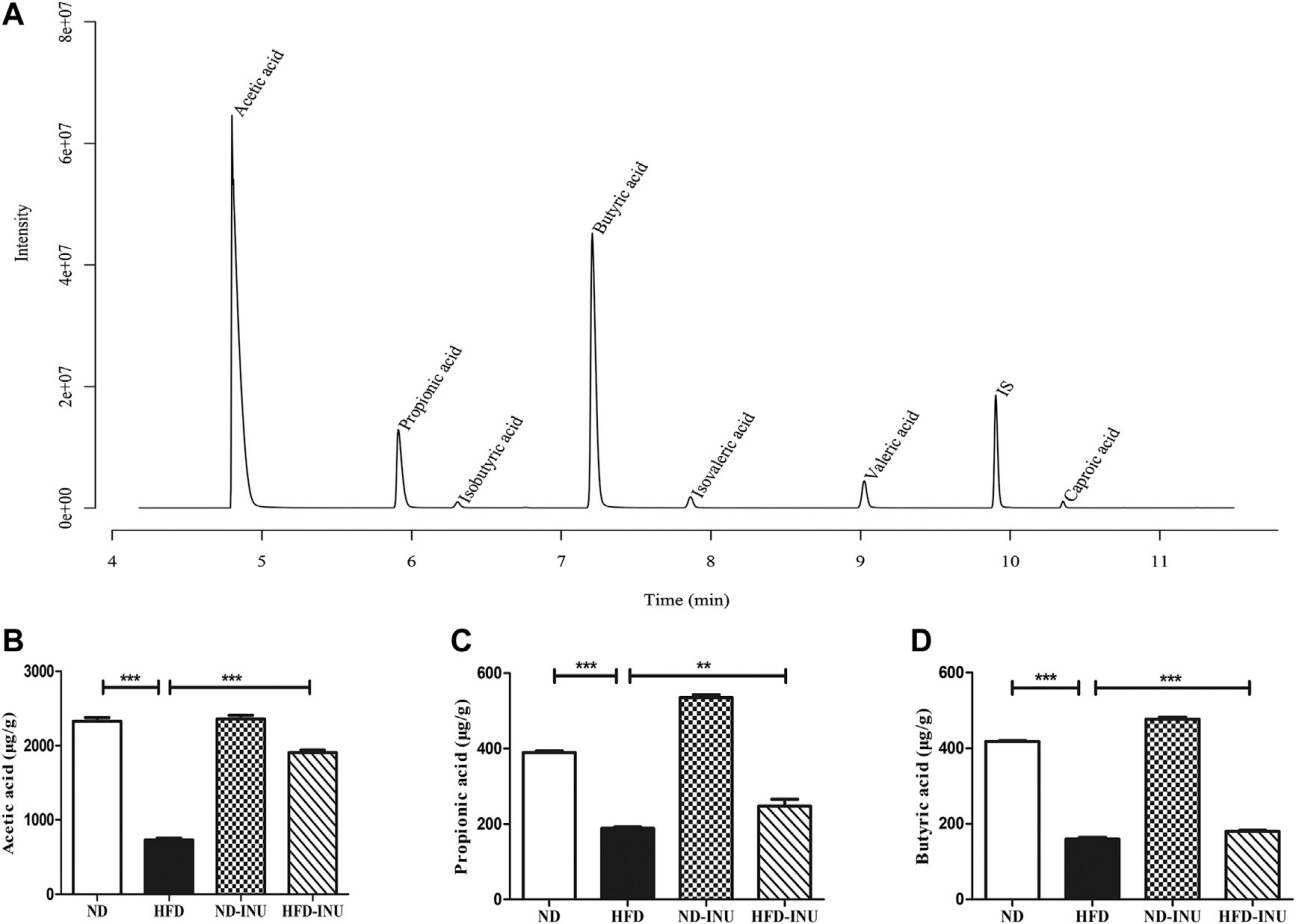
INU enhanced the concentrations of SCFAs in feces. **(A)** Sample chromatogram of mice stool. **(B)** Acetic acid. **(C)** Propionic acid. **(D)** Butyric acid. Data are expressed as mean ± SEM. *p < 0.05, **p < 0.01, ***p < 0.001.

The fecal contents of SCFAs were quantified by GC-MS primarily containing acetic acid, propionic acid and butyric acid. As shown in the chromatogram (Figure 7A), each SCFA can be distinguished clearly with a good peak shape, suggesting that the method and data were reliable. The amounts of acetic acid (*p* < 0.001, Figure 7B), propionic acid (*p* < 0.001, Figure 7C) and butyric acid (*p* < 0.001, Figure 7D) in HFD group were decreased compared with those in ND group. However, the reductions of SCFAs in HFD group were obviously elevated with INU administration (acetic acid: *p* < 0.001, propionic acid: *p* < 0.001, and butyric acid: *p* < 0.001, Figures 7B–D).

### Correlation Analysis

**FIGURE 8:**
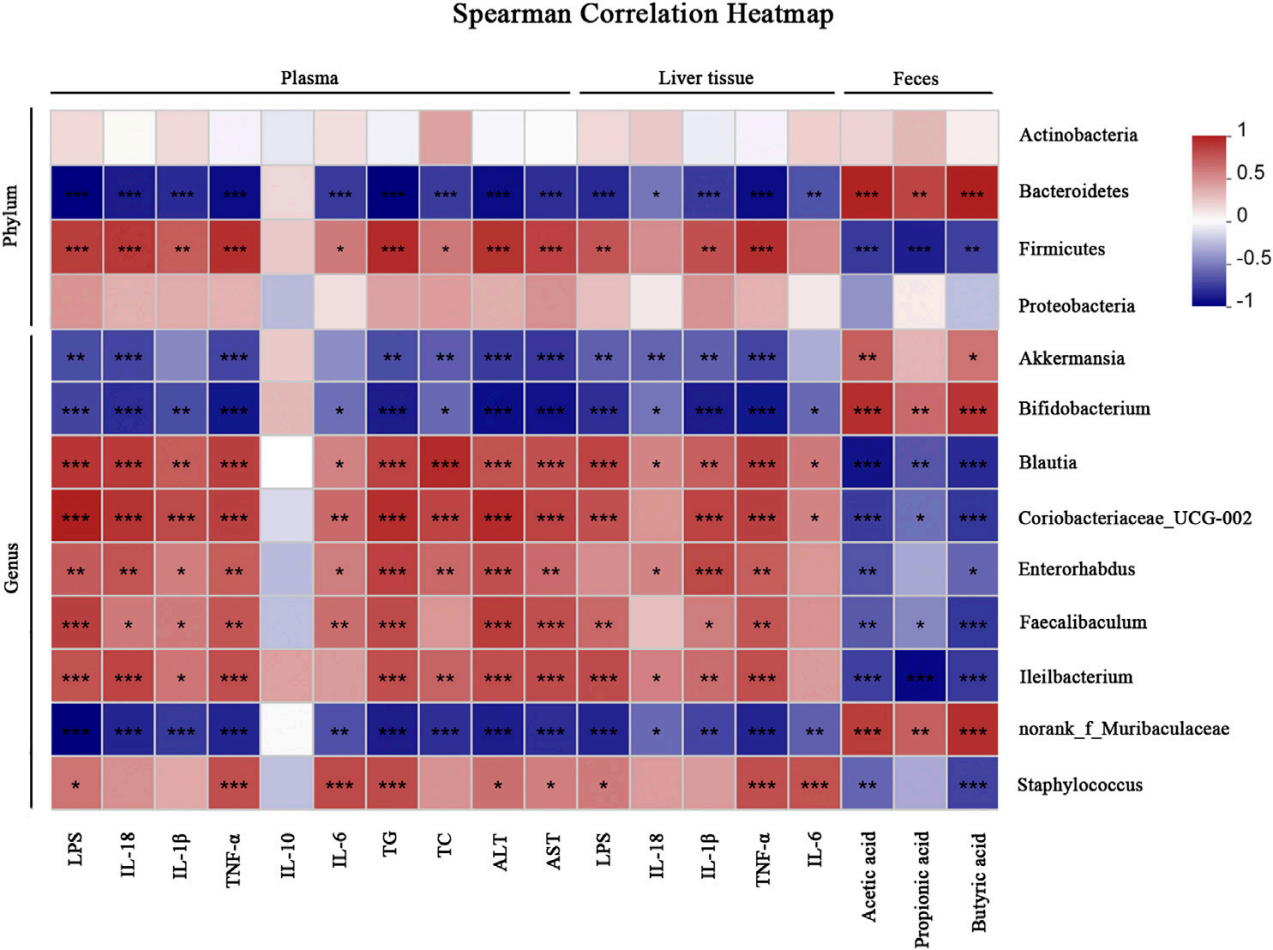
Correlation analyses between relative abundance of gut microbiota and other related indicators. *p < 0.05, **p < 0.01, ***p < 0.001.

We performed a correlation analysis among the differential bacteria, inflammation, metabolic indicators and SCFAs in NAFLD (Figure 8). The abundance of beneficial bacteria including *Bacteroidetes*, *Akkermansia* and *Bifidobacterium* exhibited positive correlations with SCFAs and IL-10, respectively. However, these beneficial bacteria were negatively correlated with metabolic and pro-inflammatory indicators (TG, TC, ALT, AST, LPS, IL-18, IL-1β, TNF-α and IL-6). Reversely, the abundance of *Proteobacteria*, *Blautia* and *Ileibacterium* were negatively correlated with SCFAs but positively associated with the above mentioned metabolic and pro-inflammatory indicators.

## Discussion

In the present study, the protective effects of dietary INU supplementation on HFD-induced NAFLD mice were determined by detecting the liver index, plasma lipid, insulin resistance, hepatic steatosis, inflammation and gut microbial community. Furthermore, the mechanism of INU on hepatic chronic inflammation of NAFLD may be related to the suppression of LPS-TLR4-Mψ-NF-κB-NLRP3 pathway, potentially providing a theoretical foundation for the prevention of NAFLD.

INU, a common prebiotics, has been proven to show an effective protection in chronic metabolic diseases (Li et al., 2019; Xue et al., 2019). INU supplementation effectively improved glucose tolerance and decreased the levels of TNF-α, IL-6 and LPS with increasing the abundance of *Bacteroides* in diabetic mice (Li et al., 2019). A recent study has demonstrated that INU consumption can drive the decrease of body mass index and improve hepatic steatosis via enriching abundances of *Akkermanisa* and *Butyricicoccus* in obese mice (Rodriguez et al., 2020). Similarly, our results showed that administration of INU evidently blocked BWs, LWs and the liver index in HFD mice while the food intake was not influenced. Furthermore, dramatical reductions of liver function (AST, ALT), lipids profile (TG, TC) and attenuation of hepatic steatosis in hepatic HE assay with INU intervention support that dietary INU can effectively attenuate the progression of NAFLD.

Insulin resistance has been thought to be the chief factor of pathogenesis in NAFLD (Day and James, 1998). Edward *et al.*(Chambers et al., 2019) found that INU supplementation improved insulin sensitivity of obese patients in a randomised clinical trial. In present study, INU intervention significantly improved glucose tolerance and insulin resistance, indicating that INU may improve insulin sensitivity in NAFLD. However, the underlying mechanism of INU on insulin resistance needs to be further investigated.

Accumulating studies have demonstrated that enterogenic endotoxemia-mediated hepatic chronic inflammation plays a critical role in the pathogenesis of NAFLD (Cani et al., 2007; Delzenne et al., 2019; Safari and Gerard, 2019). Luther *et al.*(Luther et al., 2015) found that HFD altered the composition of gut microbiota and induced the damage of integrity and permeability of gut mucosal barriers, boosting plasma LPS level. Excessive LPS translocation to liver through gut-liver axis can activate Mψs to induce inflammatory cascade reactions in NAFLD (Crawford et al., 2019; Loomba et al., 2019). To clarify the underlying mechanism of the effectiveness of INU on NAFLD, the alterations of gut microbiota and inflammatory indicators in plasma and liver tissues were further investigated. Our data revealed that levels of LPS in plasma and liver were markedly decreased with INU supplementation, indirectly demonstrating that INU may decrease translocation of LPS to systemic circulation by improving the permeability of gut barrier. The reduction of intestinal tight junction protein expression increases gut permeability, which may be the main cause of LPS leakage (Cani et al., 2009). However, the direct evidence of INU on intestinal tight junction proteins and other components of gut barriers containing intestinal mucosal immunity (such γδ^+^T cell and Treg cells) in NAFLD, still remains unclear and will be further researched.

Emerging evidences have suggested a predominant role of Mψs in low-grade inflammation of NAFLD (Alisi et al., 2017; He et al., 2017). Abnormal translocated LPS derived from gut pathogenic bacteria binds to TLR4 of Mψs to activate the transcription factor NF-κB that is responsible for inflammatory cytokine synthesis, finally leading to liver injury and hepatic steatosis (Luther et al., 2015; Loomba et al., 2019). It has been reported that under a HFD feeding in mice, an increase of activated Mψs is essential for generation of pro-inflammatory cytokines (TNF-α, IL-6 and IL-1β) as well as TLR4 expression (Leroux et al., 2012). Importantly, INU treatment could decrease TNF-α and IL-6 but increase IL-10 in diabetes mice (Li et al., 2019). In this study, we found that the proportions of F4/80^+^ cells and F4/80^+^ TLR4^+^ cells in liver were notably increased in HFD group, both of which were restored with INU intervention. Thus, concomitant with a reduction of F4/80^+^ TLR4^+^ Mψs, INU consumption also decreased the levels of pro-inflammatory cytokines (TNF-α, IL-1β, IL-18, IL-6) and elevated anti-inflammatory IL-10, suggesting that INU possessed the ability to ameliorate inflammation of NAFLD via regulating inflammatory Mψs activation and its polarization, which was similar with a study of INU in alcoholic liver disease (Yang et al., 2019). However, the exert mechanism of INU on regulating Mψ polarization in NAFLD will be researched subsequently. Intriguingly, LPS-TLR4-induced activation of NF-κB is indispensable for assembly and activation of NLRP3 inflammasome via up-regulating NLRP3 transcription by binding to the NLRP3 promoter (Qiao et al., 2012). Accelerating evidences have supported that the activation of NLRP3 inflammasome is vital for hepatic inflammation in NAFLD (He et al., 2017; Chen et al., 2018; Z. Wang et al., 2019). Increased level of NLRP3, ASC, and caspase-1 proteins expression in HFD feeding mice notably promotes the release of IL-1β and IL-18 (Zhu et al., 2018; Q. Wang et al., 2019). Consistent with these findings, our results demonstrated that HFD elevated the levels of NLRP3, ASC and caspase-1 and associated IL-1β and IL-18, whereas which was rectified with INU administration. These results indicated that protective effects of INU on NAFLD may be partially attributed to indirectly inhibiting the activation of NLRP3 inflammasome. Taken together, we highlight that INU may prevent NAFLD via suppressing LPS-TLR4-Mψ-NF-κB-NLRP3 signaling pathway through gut-liver axis.

Gut dysbiosis plays an essential role in the development of NAFLD through facilitating increased LPS to entry into circulation via portal vein, leading to inflammation and dysfunction of metabolism (Fei et al., 2020). In our study, INU supplementation elevated the overall diversity and reshaped the structure of gut microbiota in NAFLD. The two most dominant *Firmicutes* and *Bacteroidetes* in phylum level in diverse groups was consistent with previous study (Tang et al., 2018). *Firmicutes* is in favor of energy absorption by metabolizing sugar and an increased ratio of *Firmicutes* to *Bacteroidetes* can promote calories intake (Turnbaugh et al., 2006; Bai et al., 2019). Indeed, excessive calories burden is linked with the occurrence of obesity. Consistent with these studies, we found attenuation of notably increased ratio of *Firmicutes* to *Bacteroidetes* in HFD after INU supplementation, suggesting that INU positively restored the host microbial ecosystem and may be an explanation of INU alleviating HFD-induced weight gain. Moreover, with administration of INU, we found the rectification of abnormal increased *Proteobacteria* that was reported to be associated with the formation of endotoxemia (Ozkul et al., 2017; Moreira et al., 2018). Positive correlations of increased *Firmicutes* and *Proteobacteria* with pro-inflammatory (IL-6, TNF-α, IL-1β, IL-18 and LPS) as well as metabolic indicators (TG, TC, AST and ALT), demonstrated that these pathogenic bacteria contributed to the progression of NAFLD. In contrast, negative correlations of *Bacteroidetes* with pro-inflammatory and metabolic indicators suggested the beneficial potential in treatment of INU for NAFLD.

At the genus level, *Akkermansia* and *Bifidobacterium* are conductive to the reduction of LPS leakage via protecting the gut mucosal barrier function (Anhe et al., 2015; Ling et al., 2016; Grander et al., 2018). Grander *et al.* suggested that *Akkermansia* contributed to stimulating goblet cells to secret mucus and elevating the expression of gut junction proteins (Grander et al., 2018). Other studies also demonstrated that *Bifidobacterium*, commonly used as one of probiotics, improved the gut mucosal barrier function to reduce plasma LPS level for protection of NAFLD (Ling et al., 2016; Nobili et al., 2018). Intriguingly, INU supplementation increased the abundances of *Akkermansia* and *Bifidobacterium* in obese human intestinal flora (Le Bastard et al., 2019; Rodriguez et al., 2020)*.* In the present study, we also found that enrichments of *Akkermansia* and *Bifidobacterium* with INU treatment were positively correlated with SCFAs, but negatively associated with inflammatory indicators (LPS, IL-6, TNF-α, IL-1β and IL-18) and metabolic indications (TG and TC), indicating that INU could alleviate liver damage through enhancing beneficial bacteria in gut microbiota of NAFLD. Additionally, *Blautia* has been described to exhibit closely positive correlation with TG, TC, IL-6, TNF-α and IL-1β, linking to hepatic lipogenesis and obesity (Goffredo et al., 2016; Tang et al., 2018). Consistently, we found that elevated abundance of *Blautia* showed positive correlations with TG, TC and inflammatory indicators (IL-6, TNF-α, IL-1β, IL-18 and LPS), demonstrating that abnormal *Blautia* may be closely related with inflammation and metabolic dysfunction. Importantly, this abnormal increased *Blautia* could be rectified with INU supplementation, suggesting that suppression of *Blautia* may partially contribute to the effectiveness of INU on NAFLD. *Ileibacterium*, a novel member of the *Allobaculum* genus found in 2017, was associated with metabolism exerting a vigorous response to dietary changes, eventually showing protective potential for obesity (Cox et al., 2017; den Hartigh et al., 2018). We also found that an enrichment of *Ileibacterium* with HFD administration. Further analysis revealed that the bacteria showed negatively associated with SCFAs, but positively correlated with above mentioned metabolic and pro-inflammatory indications in our study. Due to limited understanding of underlying mechanism about functions and roles of above differential microbial species in NAFLD with INU treatment, the corresponding studies need to be further investigated.

Numerous evidences have suggested that SCFAs exert a critical role in regulating glycometabolism, lipid metabolism, gut barrier, and inflammation (Clemente et al., 2012; Weitkunat et al., 2017). SCFAs were gradually confirmed to exhibit anti-inflammatory and immunoregulatory functions by activating G-protein-coupled receptors (GPCRs) (Singh et al., 2014; Trompette et al., 2014; Wu et al., 2017). In our study, elevations of acetic, propionic and butyric acid with INU administration showed positive correlations with *Bacteroidetes*, *Akkermansia* and *Bifidobacterium*, but negative associations with *Firmicutes*, *Proteobacteria* as well as *Blautia*, probably attributing to alleviation of liver chronic inflammation. We speculate that an augment of SCFAs might improve hepatic inflammation through the activation of GPCRs, which will be investigated in our subsequent study. Moreover, there are other microbial metabolites that might be involved in the prevention and treatment of dietary INU on NAFLD and need to be further researched by metabonomics methodology.

In conclusion, the present study highlights that INU ameliorates NAFLD via modulating gut microbiota and suppressing LPS-TLR4-Mψ-NF-κB-NLRP3 inflammatory pathway in mice, which may potentially serve as a potent supplementary therapeutic agent against NAFLD.

## Data Availability Statement

The raw data supporting the conclusions of this manuscript will be made available by the authors, without undue reservation, to any qualified researcher.

## Author Contributions

HW, TB, FH, and SY designed and wrote the paper. TB, FH, XZ, LZ, ZW, TW, HL, and YL performed research. All authors have read and approved the final manuscript.

## Funding

This work was supported by the research and development plan of the 13th 5-years plan of Ningxia autonomous region (the major S&T projects), China (Grant No. 2016BZ02), Ningxia High School first-class Disciplines (West China first-class Disciplines Basic Medical Sciences at Ningxia Medical University), China (Grant No. NXYLXK 2017B07), Ningxia high school Top Discipline construction (Traditional Chinese Medicine Discipline, No. NXYLXK2017A06) funded project and the First class discipline construction project in Colleges and Universities of Ningxia (Grant No. NXYLXK2017A05).

## Conflict of Interest

The authors declare that the research was conducted in the absence of any commercial or financial relationships that could be construed as a potential conflict of interest.

## Glossary

NAFLD: non-alcoholic fatty liver disease
INU: inulin
ND: normal diet
HFD: high-fat diet
LPS: lipopolysaccharide
Mψ: macrophage
TLR4: Toll-like receptor four
NLRP3: nod-like receptor protein three
ASC: apoptosis-associated speck-like protein
OGTT: oral glucose tolerance test
IL-6: interleukin-6
TNF-α: tumor necrosis factor-α
IL-18: interleukin-18
IL-1β: interleukin-1β
NF-κB: nuclear factor-κB
BWs: body weights
LWs: liver weights
ALT: alanine aminotransferase
AST: aspartate aminotransferase
HOMA-IR: homeostatic model assessment of insulin resistance
TG: triglyceride
TC: total cholesterol
OTUs: operational taxonomic units
