# Supplementary material for: Inulin Exerts Beneficial Effects on Non-Alcoholic Fatty Liver Disease via Modulating gut Microbiome and Suppressing the Lipopolysaccharide-Toll-Like Receptor 4-Mψ-Nuclear Factor-κB-Nod-Like Receptor Protein 3 Pathway via gut-Liver Axis in Mice
Source: Front Pharmacol. 2020 Nov 30;11:558525. doi: 10.3389/fphar.2020.558525 (PMC7774311; doi:10.3389/fphar.2020.558525)
Supplement: Supplementary file 1 [file datasheet1.pdf]

1 . Data for Fig. 1B

Groups/weight	Week 1	Week 2	Week 3	Week 4	Week 5
ND	17.66 ± 1.03	19.55 ± 1.53	21.32 ± 2.09	24.42 ± 1.09	25.12 ± 1.22
HFD	19.03 ± 1.29	22.67 ± 2.15	23.74 ± 1.91	25.12 ± 1.87	27.52 ± 2.01
ND-INU	18.30 ± 1.02	20.06 ± 1.04	22.19 ± 1.13	23.45 ± 1.13	25.19 ± 1.13
HFD-INU	21.02 ± 1.15	22.60 ± 1.05	23.72 ± 1.32	24.98 ± 1.21	26.68 ± 1.08

Groups/weight	Week 6	Week 7	Week 8	Week 9	Week 10
ND	25.89 ± 2.63	26.55 ± 1.13	27.20 ± 1.29	27.55 ± 1.39	27.99 ± 1.69
HFD	29.43 ± 2.49	32.36 ± 2.15	35.07 ± 1.98	37.27 ± 1.51	37.98 ± 2.31
ND-INU	25.79 ± 2.32	26.46 ± 1.24	27.54 ± 1.53	27.78 ± 1.13	27.98 ± 1.71
HFD-INU	29.19 ± 2.85	30.45 ± 1.75	31.42 ± 0.87	32.72 ± 1.03	33.42 ± 1.20

Groups/weight	Week 11	Week 12	Week 13	Week 14
ND	28.25 ± 1.53	28.65 ±1.13	29.02 ± 1.59	29.87 ± 1.09
HFD	38.73 ± 1.59	39.76 ±1.85	40.47 ± 1.41	42.75 ± 1.31
ND-INU	27.79 ± 1.32	28.22 ± 1.34	28.99 ± 1.63	29.54 ± 1.13
HFD-INU	34.18 ± 1.65	35.02 ± 1.05	35.89 ± 2.08	36.32 ± 1.08

2. Data for Fig. 1C-E

Groups	Food intake	Liver wight	Liver index
ND	41.89 ± 0.75	1.09 ± 0.03	3.90 ± 0.09
HFD	36.04 ± 2.36	2.06 ± 0.15	4.47 ± 0.21
ND-INU	38.94 ± 1.44	1.06 ± 0.04	3.79 ± 0.13
HFD-INU	37.60 ± 2.00	1.60 ± 0.05	4.02 ± 0.08

3. Data for Fig. 2A, B, D-F

Groups	ALT	AST	TG	TC	Insulin	HOMA-IR
ND	42.39 ± 2.02	45.00 ± 2.55	2.29 ± 0.07	0.58 ± 0.03	2.12 ± 0.74	0.51 ± 0.20
HFD	91.19 ± 4.63	136.5 ± 5.93	4.16 ± 0.15	1.08 ± 0.10	16.84 ± 8.53	6.24 ± 1.37
ND-INU	41.79 ± 3.14	45.62 ± 2.25	2.09 ± 0.09	0.65 ± 0.05	1.04 ± 0.20	0.31 ± 0.08
HFD-INU	64.74 ± 4.43	102.70 ± 4.86	3.69 ± 0.13	0.76 ± 0.05	7.99 ± 2.04	2.16 ± 0.43

4. Data for Fig. 2C

Groups/Glucose	0min	30min	60min	90min	120min
ND	5.46 ± 0.83	24.15 ± 1.23	17.61 ± 1.89	11.35 ± 2.09	6.18 ± 1.29
HFD	14.08 ± 1.09	28.95 ± 2.75	29.85 ± 2.21	23.16 ± 2.61	13.86 ± 1.81
ND-INU	5.89 ± 0.62	19.95 ± 1.94	15.53 ± 2.13	12.52 ± 2.33	7.25 ± 2.13
HFD-INU	10.08 ± 1.35	31.02 ± 3.20	26.80 ± 2.88	16.78 ± 1.58	11.43 ± 1.08

5. Data for Fig. 3C.

Groups	NAS
ND	0.60 ± 0.40
HFD	4.60 ± 0.81
ND-INU	0.40 ± 0.24
HFD-INU	3.00 ± 0.32

6. Data for Fig. 4C, D, F.

Groups	F480	F480/TLR4	F480
ND	17.07 ± 0.80	2.06 ± 0.16	25.63 ± 2.57
HFD	29.19 ± 1.31	5.62 ± 0.30	65.83 ± 2.24
ND-INU	17.14 ± 0.84	2.19 ± 0.08	28.63 ± 2.22
HFD-INU	23.53 ± 1.25	3.55 ± 0.30	41.17 ± 2.09

7. Data for Fig. 5B-E.

Groups	NLRP3	Caspase-1	ASC	NF-κB
ND	1.18 ± 0.01	1.02 ± 0.08	0.55 ± 0.05	0.89 ± 0.11
HFD	1.57 ± 0.04	1.61 ± 0.08	1.06 ± 0.13	1.47 ± 0.04
ND-INU	1.33 ± 0.03	1.22 ± 0.02	0.66 ± 0.06	1.03 ± 0.07
HFD-INU	1.21 ± 0.03	1.27 ± 0.06	0.57 ± 0.04	1.22 ± 0.03

8. Data for Fig. 5 F, G, J, N, L, P

Groups	IL-1β	IL-18	LPS	TNF-a	IL-6	IL-10
ND	24.73 ± 2.99	17.28 ± 1.99	0.13 ± 0.00	5.91 ± 0.31	2.72 ± 0.17	13.97 ± 1.02
HFD	41.95 ± 3.31	40.82 ± 2.94	0.27 ± 0.01	12.07 ± 0.70	3.85 ± 0.35	12.35 ± 0.95
ND-INU	28.43 ± 2.77	21.13 ± 2.08	0.12 ± 0.01	6.00 ± 0.45	2.03 ± 0.36	14.60 ± 1.08
HFD-INU	31.16 ± 2.52	31.71 ± 1.65	0.20 ± 0.01	8.82 ± 0.37	2.66 ± 0.21	17.70 ± 0.69

9. Data for Fig. 5 H, I, K, O, M

Groups	Liver IL-1β	Liver IL-18	Liver LPS	Liver TNF-a	Liver IL-6
ND	4.74 ± 0.25	5.18 ± 0.32	0.20 ± 0.00	1.56 ± 0.13	0.24 ± 0.03
HFD	7.06 ± 0.64	8.30 ± 1.14	0.31 ± 0.02	5.35 ± 0.22	0.46 ± 0.05
ND-INU	4.57 ± 0.53	5.56 ± 0.13	0.19 ± 0.01	1.28 ± 0.18	0.14 ± 0.04
HFD-INU	4.97 ± 0.32	5.39 ± 0.50	0.25 ± 0.00	3.17 ± 0.18	0.25 ± 0.03

10. Data for Fig. 7B-D

Groups	Acetic acid	Propinic acid	Butyric acid
ND	2330.00 ± 46.05	389.70 ± 4.69	417.80 ± 2.59
HFD	734.00 ± 22.03	188.50 ± 3.63	159.00 ± 5.22
ND-INU	2385.00 ± 49.05	535.10 ± 8.16	476.60 ± 5.78
HFD-INU	1906.00 ± 33.35	247.90 ± 17.94	180.00 ± 2.84
